# Unraveling the flavor formation mechanism during yak sour meat fermentation: Integrated multi-omics and machine learning approaches

**DOI:** 10.1016/j.fochx.2026.103866

**Published:** 2026-04-18

**Authors:** Peiting Zhang, Chenshuo Wang, Peiyi Wang, Zilin Shen, Jiazhuo Gao, Shihong Fang, Chenglin Zhu, Luca Laghi

**Affiliations:** a College of Pharmacy and Food, Southwest Minzu University, Chengdu, Sichuan 610041, China; b College of Computer Science and Artificial Intelligence, Southwest Minzu University, Chengdu, Sichuan 610041, China; c Key Laboratory of Research and Application of Ethnic Medicine Processing and Preparation on the Qinghai Tibet Plateau, Southwest Minzu University, Chengdu, Sichuan 610041, China; d Sichuan Zoige Alpine Wetland Ecosystem National Observation and Research Station, Southwest Minzu University, Chengdu 610041, China; e Department of Agricultural and Food Sciences, University of Bologna, Cesena 47521, Italy

**Keywords:** Bacterial diversity, Correlation bacteria-flavor, Intelligent sensory, Yak sour meat

## Abstract

Fermentation determines the physicochemical properties and flavor characteristics of sour meat. However, research on yak sour meat fermentation remains limited, compared to pork's counterpart. This study combined physicochemical analysis, intelligent sensory techniques, gas chromatography - ion mobility spectrometry (GC-IMS), 16S rRNA sequencing and machine learning (ML) to explore bacterial dynamics and flavor development during a 45-day fermentation procedure. *E*-sensing distinguished stage-specific sensory profiles, with day-45 samples showing enhanced umami and sweetness. GC-IMS identified 42 volatiles, with 3-methylbutanal isomers, acetic acid and *α*-terpinolene increasing and 2-butoxyethanol, hexanal-D decreasing during fermentation. Bacterial diversity declined, with *Staphylococcus* and *Lactobacillus* dominating, and *Lactobacillus* was confirmed as the key genus for flavor formation. Fermentation caused a pH reduction, and a hardness elevation, accompanied by modifications to the color's profile. ML models demonstrated high predictive performance: Support Vector Machine (SVM) achieved 100% accuracy in classifying fermentation stages using *E*-nose/E-tongue data, while k-Nearest Neighbors (*k*−NN) optimally differentiated stages based on GC-IMS volatile profiles. This study elucidates the interplay between microbial succession and flavor evolution in yak sour meat, establishing a scientific foundation for optimizing fermentation protocols and enhancing product quality control.

## Highlights

• Multi-omics revealed Lactobacillus-driven flavor dynamics in yak sour meat fermentation.
• GC-IMS identified 42 volatiles, esters dominated T45's umami/sweetness enhancement.
• E-nose/tongue discriminated fermentation stages with sensory specificity.
• Acidification suppressed spoilage Pseudomonas, ensuring safety.
• Microbial-flavor correlations guide optimized fermentation protocols.
• For fermentation stage prediction, SVM excels with E-nose/tongue data, k-NN with GC-IMS data.

## 1 Introduction

Sour meat is a Chinese fermented traditional food with a history spanning more than two millennia. It is mainly produced in Yunnan, Hunan, and Guizhou provinces. It is produced by mixing pork, rice noodles, salt, and seasonings (e.g., chili powder, Sichuan pepper powder). The mixture then undergoes fermentation under sealed, anaerobic conditions for one to two months. Its characteristics include a firm texture, pronounced sour taste, rich ester aroma, a fatty but not greasy mouthfeel, and high nutritional value due to being rich in probiotics and postbiotic components (Cheng et al., 2024; Lv et al., 2021). During sour meat fermentation, enzymatic catalysis by endogenous proteases and microbial activity (e.g., *Lactobacillus* and *Staphylococcus*) through hydrolysis of lipids, autoxidation, proteolysis, and amino acid/carbohydrate metabolism—to generate diverse flavor compounds, namely esters, alcohols, acids, and free amino acids (Zeng et al., 2021). In addition, fermentation markedly alters the physicochemical properties of sour meat (e.g., pH, color and texture), closely linked to bacterial community activity (Lv et al., 2021; Lv, Li, et al., 2019; Lv, Yang, et al., 2019). Typical changes include lower pH (Cheng et al., 2024; Liu et al., 2024; Zang et al., 2025) and springiness and increased hardness and chewiness (Lan et al., 2025; Shang et al., 2024).

Apart from fermentation, meat source can also influence the physiochemical features and flavor profiles of sour meat (X. Zhao et al., 2023). For example, b* value decreased significantly during goose sour meat fermentation but increased significantly during pork sour meat fermentation. (Hu et al., 2022; Lan et al., 2025; Stadnik et al., 2022). However, existing research has mainly focused on pork sour meat. Studies on flavor formation and bacterial succession during yak sour meat fermentation remain limited. Compared to the other meat, yak meat, originating from the Qinghai-Tibet Plateau, can be considered as a nutritionally richer protein source, containing 20–23% protein and approximately 5% fat (S. Li et al., 2023). Moreover, the flavor profile of yak meat differs from that of other meats. Because yak meat contains more unsaturated fatty acids, its flavor arises mainly from lipid oxidation, which produces characteristic volatiles such as heptanal, nonanal, hexanal, 1-octen-3-ol, and octanal. These components endow yak meat with both the aroma of beef tallow and the fragrance of green grass (Y. Fu et al., 2022; Han et al., 2020; Huang, Dong, et al., 2022). Therefore, investigating the dynamic changes of bacteria communities during yak sour meat fermentation and their impact on flavor compound formation holds significant theoretical and practical importance.

To date, existing studies on yak meat flavor have predominantly focused on fresh yak meat, employing advanced analytical techniques such as gas chromatography–ion mobility spectrometry (GC-IMS), gas chromatography–mass spectrometry (GC–MS), metabolomics, and lipidomics to investigate the effects of oxidation(Huang, Sun, et al., 2022), aging (X. Wang et al., 2025), feeding regimes (Wang, Shi, et al., 2026), types and cuts (Li, Zhang, et al., 2024; Zhang, Zeng, et al., 2024), as well as packaging methods (Guo et al., 2026) on volatile compound profiles. Comparative studies on sour meat from different meat sources have also been conducted using multi-omics approaches (Lan et al., 2026). However, systematic investigations specifically targeting the dynamic bacterial succession and flavor formation mechanisms during the fermentation of yak sour meat remain extremely scarce, with fewer than five published studies available. Moreover, no study has yet integrated multi-platform flavor analysis (GC-IMS, *E*-nose, E-tongue) with 16S rRNA sequencing and machine learning to comprehensively characterize the microbe–flavor interplay throughout the yak sour meat fermentation process.

To fulfill the above requirements, GC-IMS is considered as one of the best choices for flavor analysis (Zhang et al., 2024), E-nose and E-tongue are optimal for aroma pattern recognition (Q. Zhang et al., 2023) and taste feature discrimination (Gao et al., 2020; Jiang et al., 2021), respectively. In addition, parallel microbial insights are achieved through 16S rRNA amplicon sequencing. The integration of metabolomic and metagenomic information enables the comprehensive mapping of microbial-flavor relationships, of crucial importance in traditional products based on meat upon fermentation. Examples of the success of this multifaceted approach can be found in the work of Wen, who recently revealed an inverse correlation between drying duration and microbial diversity in Tibetan yak jerky (Wen et al., 2021), suggesting its utility in tracking fermentation-driven biodiversity shifts.

Recent advances in machine learning (ML) have extended its use to analyzing volatile organic compounds for food aroma profiling. By building models to identify key flavor constituents, ML provides superior accuracy and efficiency to conventional statistical methods in handling complex, high-dimensional, and nonlinear relationships (Xiong et al., 2023). Common techniques include artificial neural networks (ANNs), support vector machines (SVMs), and random forests (RFs) (Goyal et al., 2024) ANNs are particularly suitable for modeling intricate nonlinear patterns (M. Zhu et al., 2025); SVMs maintain strong generalization and prediction accuracy with limited samples through structural risk minimization (Y. Wu et al., 2025); and RFs resist overfitting, handle high-dimensional data, and improve classification via ensemble learning (Breiman, 2001). These applications demonstrate how ML enhances analytical precision in flavor research. Notably, Yang et al. (C. Yang et al., 2025) combined untargeted metabolomics with ML to differentiate rare honeys by botanical origin using flavonoid profiles. Li et al. (Li et al., 2024) employed GC-IMS and headspace solid-phase microextraction (HS-SPME) / GC–MS to track volatile dynamics during grape tea processing, where a random forest model identified key flavor markers. Furthermore, Wang et al. (Wang, Wang, et al., 2026) integrated multi-platform analyses-including GC–MS, GC-IMS, and comprehensive two-dimensional gas chromatography–mass spectrometry (GC × GC–MS)—with ML to characterize cherry juice volatiles, finding the Naive Bayes Model (NBM) achieved optimal classification and prediction performance. Despite advances in fermented meat research, no study has yet systematically integrated multi-platform flavor analysis—including GC-IMS, *E*-nose, and E-tongue—with 16S rRNA sequencing and machine learning to characterize the dynamic flavor formation and bacterial succession during yak sour meat fermentation. To address this critical gap, the present study establishes a novel analytical framework that combines physicochemical, sensory, and omics data to elucidate the interplay between microbial dynamics and flavor evolution in this traditional product, thereby providing a methodological reference for quality control in fermented meats.

To achieve these goals, this study aimed to: (1) Systematically characterize the physicochemical properties of yak sour meat, with emphasis on dynamic mechanisms underlying fermentation-induced changes in pH, color parameters (*L*^∗^, *a*^∗^, and *b*^∗^), and textural properties (e.g., hardness, springiness, chewiness); (2) Discriminate aroma/taste profiles and identify volatile compounds during fermentation using intelligent sensory techniques and GC-IMS; (3) Analyze bacterial community succession via 16S rRNA amplicon sequencing; (4) Integrate physicochemical indices, GC-IMS metabolomics, and 16S rRNA amplicons sequencing data to investigate relationships among bacterial communities, pH, and volatile compounds through Pearson correlation analysis. (5) Evaluate the feasibility and performance of ML models in accurately classifying fermentation stages based on multi-source flavor data. Ultimately, this work seeks to elucidate these mechanisms, which is expected to shed light on optimizing the overall fermentation process and enhancing the quality of yak sour meat.

## 2 Materials and methods

### 2.1 Preparation of the samples

Yak *longissimus lumborum* muscles were purchased from Yipin Fresh Supermarket (Chengdu, China) and transported to the laboratory in ice boxes, where visible connective tissue and fat were removed before further processing. The food ingredients were obtained from Walmart Stores (Shuangliu, Chengdu, China), including salt, pepper, glutinous rice and glutinous rice powder. Following the method described by Zhao et al. (X. Zhao et al., 2023), meat sample was chopped (3 cm × 5 cm × 0.6 cm), mixed with salt (5% *w*/w), and marinated for 2 h at 4 °C. In parallel, the glutinous rice was toasted to a golden brown before being ground until coarse particles were obtained, passing through a 30-mesh sieve. The marinated meat was then combined with 15% pepper (*w*/w), 25% glutinous rice powder (w/w) and 25% steamed glutinous rice (w/w). The mixture (200 g) was packed into airtight jars, placed at a temperature of 20 ± 2 °C for fermentation. All jars were opened at the same time for sampling when it reached time points, and then were fully sealed. Sour meat samples from the same one jar were considered as an experimental replicate, and three measurements were performed for every experimental replicate.

### 2.2 Physicochemical analysis

#### 2.2.1 Textural analysis

According to the previous method (Lv et al., 2023), a texture analyzer (TA-XT Plus, Stable Micro Systems, UK) was used to analyze springiness, hardness, chewiness and cohesiveness. Parameters for measurement were as follows: probe type, P/50; velocity of 2.0 mm/s (pre-test, test, and post-test); first and second pressing separated by 5 s; 10 g of trigger force.

#### 2.2.2 Determination of color parameters

According to a previously outlined method (Hu et al., 2024), color parameters a* (redness) and b* (yellowness) and L* (lightness) of yak meat samples were determined with a Konica Minolta CR-400 colorimeter (Tokyo, Japan). Measurements were taken at three distinct locations on the cross-sectional surface after exposing samples to ambient air at 4 °C for 30 min. The instrument was calibrated with a ceramic white plate prior to measurement.

#### 2.2.3 pH analysis

Two grams of the crushed sample were introduced in a 50 mL sample tube together with 20 mL of distilled water. Following a previously set-up method (Lv et al., 2019), homogenization was then applied three times at 15 s intervals, using a T25 Digital ULTRA-TURRAX homogenizer (IKA®, Germany) set at 3000 rpm. The pH of the resulting homogenate was then measured using a pH meter (Five Easy Plus FE28, Mettler Toledo, China).

### 2.3 Intelligent sensory analysis

#### 2.3.1 *E*-nose analysis

E-nose analysis was performed with a system Fox 4000 (Alpha MOS, France), featuring 18 sensors based on metal oxides. Table S1 provides the key details on each sensor. Following a previous method (Zhou et al., 2024), 0.25 g meat was introduced in a bottle with 10 mL of internal volume, warmed for 5 min at 70 °C and then injected manually in the system. The sample detection time was 120 s. A cleaning time of 240 s avoided interferences from previous measurements. Every sample was tested ten times, and three stable values were used for the subsequent data analysis.

#### 2.3.2 E-tongue analysis

The measurements, following a previously described method (Zhang et al., 2024), were conducted with an Alpha MOS (France) system *α*-ASTREE. The apparatus has a sixteen positions autosampler and sensors tailored for saltiness (CTS), sourness (AHS), sweetness (ANS), umami (NMS), bitterness (SCS). The system is also equipped with two electrodes for reference, designated as CPS and PKS. To extract taste compounds, each sample of approximately 20 g was mixed with demineralized water (200 mL). After 10 min of centrifugation at 2265 ×*g* and 4 °C, the aqueous phase (100 mL) underwent the analysis. Each measurement cycle lasted 120 s, stirring the samples at 60 rpm and then cleaning the analysis chamber for 30 s with demineralized water. Average values measured in the 100–120 s interval were utilized for subsequent analysis.

### 2.4 GC-IMS analysis

Volatile compounds were characterized by GC-IMS (FlavourSpec®, G.A.S., Germany), according to the method outlined by Yang et al. (Yang, Li, & Guo, 2024). Samples of 0.25 g were equilibrated at 50 °C for 10 min in a sample vial, then extracted at 65 °C using a system for headspace sampling. IMS was performed using an MXT-WAX column (30 m × 0.53 mm × 1 μm, Restek, United States). The flow rate of the carrier gas (N_2_) was 5 min at 2 mL/min, 10 min at 10 mL/min, 5 min at 15 mL/min, 10 min at 50 mL/min, and 10 min at 100 mL/min. An external reference made by n-ketones C4-C9 was employed to calculate the index of retention (RI) of the various compound. The identification of the volatile substances was obtained through the comparison of their retention indices and drift times with the library equipping the GC-IMS instrument.

### 2.5 Bacterial diversity analysis

Each sample of approximately 0.25 g was placed under aseptic conditions in sterile cryovials, where they were stored at −80 °C after a fast-freezing step with liquid N_2_. The bacterial communities' genomic DNA was extracted by taking advantage of a E.Z.N.A.® (Omega Bio-tek, Norcross, USA) DNA Kit for soils. The DNA served as a template for PCR amplification of the V3-V4 variable region of the 16S rRNA gene. The amplification was obtained with the upstream primer 338F (5’-ACTCCTACGGGAGGCAGCAG-3′), with a Barcode sequence, and with the downstream primer 806R (5’-GGACTACHVGGGTWTCTAAT-3′). A 2% agarose gel was used to recover the products of the PCR, then a PCR Clean-Up Kit (Q. Zhang, et al., 2024) was used to purify them. Purified amplicons were combined equimolarly and sequenced in a paired-end mode on an MiSeq PE300/PE250 platform from Illumina (Illumina, San Diego, USA). Following the protocols of Majorbio Bio-Pharm Technology Co. ltd. (China). Fastp procedure (ver. 0.20.0) was used to demultiplex and then quality-filter the raw 16S rRNA gene sequencing reads. The reads were then merged by FLASH version 1.2.7. The optimized sequences were further processed by DADA2 to remove PCR amplification and sequencing errors, leading, in each sample, to real Amplicon Sequence Variants (ASV) (Callahan et al., 2016).

### 2.6 Model building and validation for ML

ML analysis was implemented in Python 3.9 with scikit-learn, SciPy, NumPy, and Matplotlib. The overall workflow consisted of two main phases: data preprocessing followed by model validation. Initially, raw features were log-transformed to stabilize variance and were subsequently standardized via *Z*-score normalization (mean = 0, SD = 1). The resulting processed dataset was then used to train six distinct classifiers: logistic regression, linear SVM, radial basis function SVM (RBF SVM), k-nearest neighbors (k-NN), linear discriminant analysis (LDA), and naive Bayes. Due to the small sample size, stratified 3-fold cross-validation was applied to maintain class distributions. Model performance was measured using accuracy, macro-averaged F1-score, Cohen's kappa, and the Matthews correlation coefficient (MCC), all of which were derived from the confusion matrix parameters: true positive (TP), true negative (TN), false positive (FP), and false negative (FN). N is the number of samples. K is the total number of categories. TP and TN represent the samples correctly identified in their respective categories. Accuracy was defined by Eq.1:

(1) $$\text{Accuracy}=\frac{\mathrm{TP}+\mathrm{TN}}{N}$$

The macro-averaged F1-score is computed by first calculating the F1-score for each class i (Eq.2) and then averaging across all K classes (Eq.3):

(2) $$F1_{i}=\frac{2\cdot TP_{i}}{2\cdot TP_{i}+FP_{i}+FN_{i}}$$

(3) $$F1_{\text{macro}}=\frac{1}{K}\sum_{i=1}^{K}F1_{i}$$

Cohen's kappa κ was defined by Eq.4:

(4) $$\kappa =\frac{p_{o}-p_{e}}{1-p_{e}}$$

where $p_{o}$is the observed agreement and $p_{e}$is the expected agreement under the assumption of random classification. The MCC was defined by Eq.5:

(5) $$\mathrm{MCC}=\frac{c\cdot s-\sum_{k=1}^{K}p_{k}t_{k}}{\sqrt{\left(s^{2}-\sum_{k=1}^{K}p_{k}^{2}\right)\left(s^{2}-\sum_{k=1}^{K}t_{k}^{2}\right)}}$$

Where s is the total number of samples; c is the number of correctly classified samples; $p_{k}$ is the number of predictions for class k; and $t_{k}$ is the number of true instances for class k. Finally, comparative performance across all classifiers was summarized in a bar chart, and the best-performing model was further examined through a confusion matrix to visualize its predictive patterns.

### 2.7 Statistical analysis

Data mining was performed in R language. Data were normalized by following the method described by Box and Cox (Box & Cox, 1964). Then, ANOVA with Tukey's test *(P* < 0.05) identified significant differences among groups. As suggested by previous study (C. Zhu et al., 2022), a principal component analyses in its robust mode (rPCA) were employed to look for overall trends underlying the data. This was done on the mean responses from *E*-tongue and from E-nose, and on the signals from GC-IMS. The outcome of each rPCA model, consisted in a score plots and in a correlation plot, according to Pearson. The two plots were tailored to visualize, respectively, the distribution of data and correlations between model components and variables. On the Majorbio platform (https://www.majorbio.com), non-metric multidimensional scaling (NMDS) was used to assess bacterial community succession during fermentation, in combination with effect size of Linear discriminant analysis effect size (LEfSe). Correlations among pH, volatile compound and bacterial communities were calculated using the online tool Metware Cloud (https://cloud.metware.cn).

## 3 Results

### 3.1 Physicochemical properties

Changes in the physicochemical properties of yak sour meat during fermentation are shown in Table 1.

**Table 1:** Changes in texture, color and pH in the sour meat of yak, at different fermentation stages.

	Day 0	Day 15	Day 30	Day 45
Hardness (g)	1.01 × 10^2^ ± 31.50^b^	2.44 × 10^2^ ± 46.10^b^	2.19 × 10^2^ ± 42.50^b^	8.31 × 10^2^ ± 2.32 × 10^2a^
Chewiness (g)	43.60 ± 8.54^b^	1.24 × 10^2^ ± 32.40^b^	90.30 ± 28.20^b^	4.18 × 10^2^ ± 1.46 × 10^2a^
Cohesiveness	0.65 ± 5.43 × 10^-2a^	0.72 ± 6.22 × 10^-2a^	0.67 ± 3.33 × 10^-2a^	0.75 ± 6.93 × 10^-2a^
Springiness	0.68 ± 4.06 × 10^-2a^	0.70 ± 3.90 × 10^-2a^	0.61 ± 9.04 × 10^-2a^	0.68 ± 0.11^a^
*a*^*⁎*^	4.62 ± 1.76^c^	10.60 ± 1.33^b^	14.60 ± 2.65^a^	9.90 ± 2.08^b^
*b*^⁎^	5.74 ± 1.61^c^	11.10 ± 1.05^b^	14.90 ± 2.71^a^	10.50 ± 1.99^b^
*L*^⁎^	44.90 ± 1.05^b^	47.80 ± 0.04^a^	49.40 ± 1.60^a^	47.60 ± 1.31^a^
pH	5.59 ± 7.51 × 10^-2a^	4.36 ± 6.24 × 10^-2b^	4.01 ± 1.15 × 10^-2c^	3.98 ± 2.08 × 10^-2c^

Values are presented as mean ± sd. Significant differences (*P* < 0.05) are highlighted by different superscript letters.

Texture attributes, including hardness, chewiness, cohesiveness, and springiness, are crucial parameters for assessing the edible quality of yak sour meat. In detail, the hardness and chewiness values in Day 45 samples significantly differed from those in the other groups (*P* < 0.05), while not significant changes occurred in springiness and cohesiveness. Similarly, color serves as a fundamental quality indicator influencing consumer acceptance. Notably, color parameter a* and b* increased significantly from Day 0 to Day 30 but decreased significantly from Day 30 to Day 45 (*P* < 0.05). The L* values of the samples in the Day 0 group were significantly lower than those of the other groups (*P* < 0.05). In addition, pH values significantly decreased during fermentation.

### 3.2 Intelligent sensory discrimination

#### 3.2.1 Aroma discrimination by *E*-nose

**Fig. 1:**
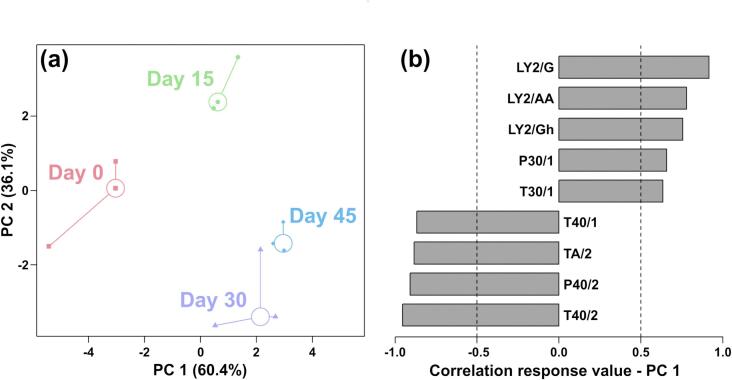
Score plot (a) and loading plot (b) of an rPCA model based on the response data of E-nose sensors.

E-nose results during fermentation of yak sour meat are shown in Fig. 1. In the rPCA score plot (Fig. 1(a)), PC1 accounted for 60.4% of the total variance, effectively discriminating the four sample groups. Samples from Day 0 and Day 15 clustered in the left quadrant (negative PC1 values), while those from Day 30 and Day 45 occupied the right quadrant (positive PC1 values), showing that the aroma profile of yak sour meat was significantly influenced by fermentation. Furthermore, as shown in Fig. 1(b), sensors LY2/G, LY2/AA, LY2/Gh, P30/1, and T30/1 exhibited elevated response toward samples from Day 30 and Day 45. Conversely, sensors T40/1, TA/2, P40/2, and T40/2 showed higher response values for samples collected at Day 0 and Day 15.

#### 3.2.2 Taste discrimination by E-tongue

**Fig. 2:**
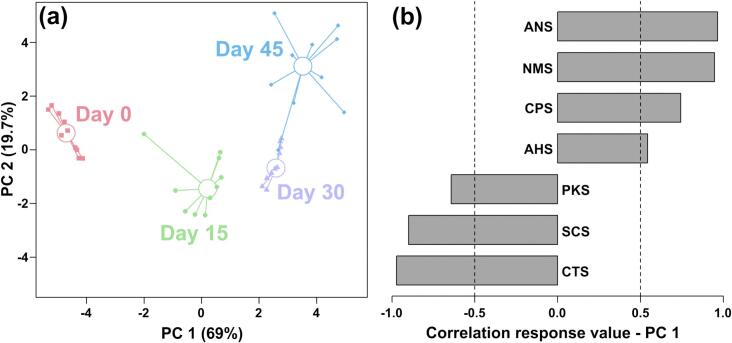
Score plot (a) and loading plot (b) of an rPCA model based on the response data of E-tongue sensors.

E-tongue analysis was employed to objectively evaluate taste characteristics during yak sour meat fermentation (Fig. 2). PC1 in the rPCA score plot accounted for 69.0% of the total variance explained by the model, while PC2 explained 19.7%, collectively differentiating the four fermentation stages (Fig. 2(a)). Samples from Day 0 clustered within the negative PC1 quadrant, contrasting with Day 30 and Day 45 samples in the positive quadrant, indicating a significant flavor profile evolution. Furthermore, Fig. 2(b) shows that after fermentation, the response values of the sensors ANS, NMS, and AHS increased significantly, while the response values of the CTS and SCS sensors decreased.

### 3.3 Identification and classification of volatile compounds based on GC-IMS

**Fig. 3:**
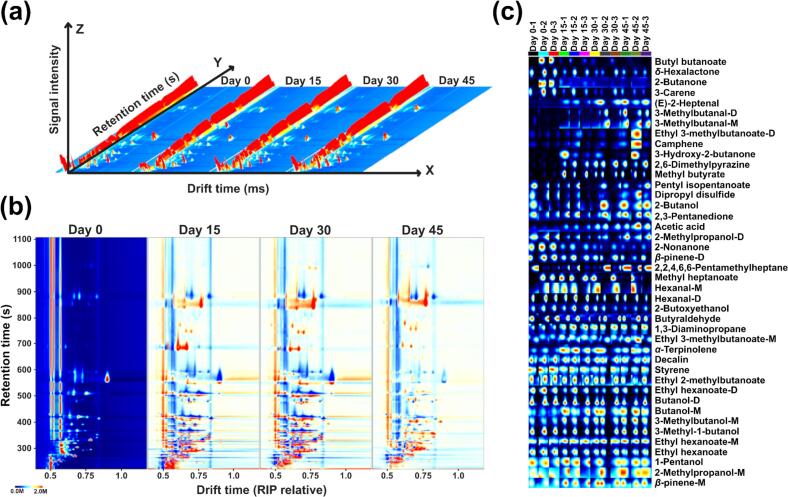
Results of GC-IMS analysis of yak sour meat at different fermentation stages. (a) Morphology plot in three dimensions. (b) 2D difference plots using as a reference the spectra from the samples collected at day 0. (c) Representation as fingerprint plot of the concentration's difference of volatile compounds among the four groups.

GC-IMS database matching was employed to identify the volatile flavor compounds during the fermentation of yak sour meat. Fig. 3(a) shows the three-dimensional GC-IMS spectra of yak sour meat, which directly indicates the differences in flavor compounds. The X, Y and Z axes correspond to drift time, retention time and peak intensity, respectively. The red vertical line on the left side indicates the reactive ion peaks, while each dot corresponds to a specific compound. The color depth symbolizes the peak intensity of the compound, with blue indicating peaks of relatively low intensity and red indicating peaks of higher intensity. Fig. 3(b) provides a detailed comparison of the groups, highlighting specific compounds with retention times (RT) between 300 and 1100 s and ion drift times between 1.0 and 1.5 ms. Fingerprint analysis (Fig. 3(c)) displays sample-specific volatile profiles, with rows representing compounds, columns indicating fermentation stages. A total of 42 volatile compounds were identified in yak sour meat, including esters (11), alcohols (9), ketones (4) and aldehydes (6), and the specific information of volatile compounds is shown in Table S2.

**Fig. 4:**
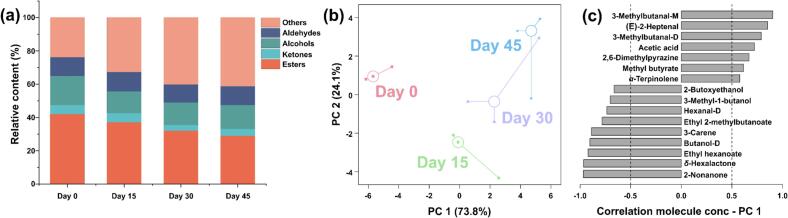
rPCA model based on GC-IMS data. (a) Histograms plot. (b) Score plot showing the samples collected at day 0 with squares, at day 15 with circles, at day 30 with triangles and at day 45 with diamonds. Wide, empty circles indicate the median of each group. (c) Loadings, expressed as correlations of molecules' concentrations with their importance in determining PC 1.

Fig. 4(a) shows that esters were predominant in the yak sour meat during fermentation, with contributions from alcohols and aldehydes. In the rPCA score plot (Fig. 4(b)), PC 1 explained 73.8% of the total variability among the samples, effectively differentiating the four experimental groups. Day 0 group showed negative scores along PC1, while Day30 and Day45 showed positive scores. Pearson correlation plots of loadings (Fig. 4(c)) revealed that seven volatile compounds were significantly positively correlated with PC1 including 3-methylbutanal-M, (E)-2-heptenal, 3-methylbutanal-D, acetic acid, 2,6-dimethylpyrazine, methyl butyrate and *α*-terpinolene. In contrast, 2-butoxyethanol, 3-methyl-1-butanol, hexanal-D, ethyl 2-methylbutanoate, 3-carene, butanol-D, ethyl hexanoate, *δ*-hexalactone and 2-nonanone showed a significant negative correlation with PC1.

### 3.4 Bacterial diversity and components

#### 3.4.1 Bacterial community diversity

*α*-Diversity reflects the richness and diversity of bacterial communities. Ace, Chao and Sobs indices are employed to assess the richness of bacteria in samples, and Shannon and Simpson indices are used to estimate bacterial diversity (Shen et al., 2021).

**Fig. 5:**
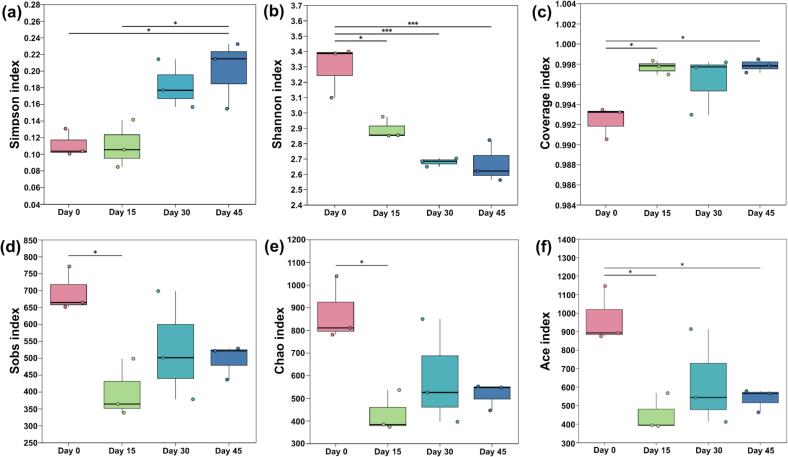
*α*-diversity index of samples during yak sour meat fermentation. “*” *P* < 0.05, “**” 0.001 < *P* ≤ 0.01 and “***” *P* ≤ 0.001.

Analyzing bacterial diversity is an effective method to assess the composition of microbial communities during yak sour meat fermentation. The *α*-diversity indices of the bacterial community, mainly including Simpson, Shannon, Coverage, Sobs, Chao,

and ACE, were used to assess species richness as shown in Fig. 5(a-f). The index of coverage of all sour meat samples was higher than 0.99, suggesting that the data of sequencing had high integrity and accuracy. The Sobs, ACE, and Chao indices for sour meat samples decreased along fermentation, indicating a decline in the number, richness, and overall diversity of the bacterial community. Focusing on Shannon index, considered an index of richness and even distribution within the bacterial community of the samples, it can be noted that its highest values were found in the Day 0 group, indicating that the bacterial community at the beginning of the fermentation exhibited the greatest level of diversity.

**Fig. 6:**
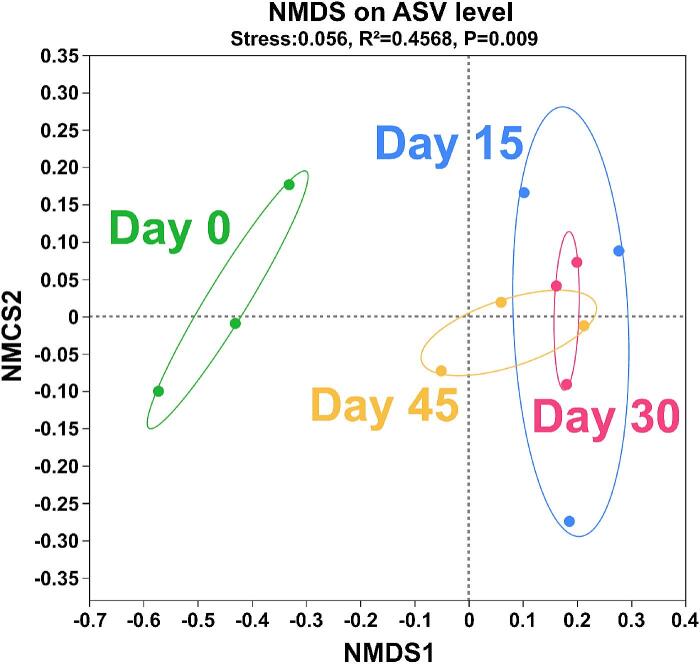
NMDS analysis of bacterial communities on ASV during yak sour meat fermentation.

*β*-diversity refers to the diversity of species composition or the rate of species replacement along environmental gradients among different communities. The similarity in community structure between the different groups was reflected by NMDS analysis. The microbial profiles of Day 15, Day 30 and Day 45 were more similar, as shown in Fig. 6. The microbial profiles of the samples from the Day 0 group can be clearly distinguished from the microbial profiles of the samples from the Day 45 group. Indicating that the community composition highly varied during fermentation.

#### 3.4.2 Bacterial composition

**Fig. 7:**
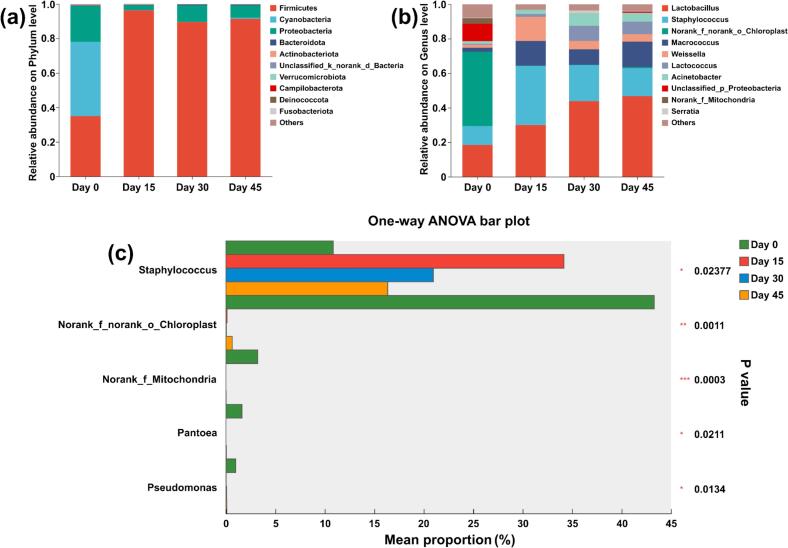
Columnar cumulative plots of bacterial community abundance at the levels of phylum (a) and genus (b). Bar graph of differences between groups in mean relative abundance of the same species (c), different colors indicate different subgroups. “*” *P* < 0.05, “**” 0.001 < *P* ≤ 0.01 and “***” *P* ≤ 0.001.

Bacterial taxonomic composition was analyzed at phylum and genus levels. At the phylum level (Fig. 7(a)), ten bacterial phyla with relative abundance >1% were identified in fermented samples, predominantly *Firmicutes*, *Cyanobacteria*, and *Proteobacteria*. During fermentation, *Firmicutes* abundance increased markedly, while *Cyanobacteria* decreased progressively. Similarly, ten genera exceeded 1% relative abundance at the genus level (Fig. 7(b)). *Lactobacillus* and *Staphylococcus* emerged as dominant genera throughout fermentation. Specifically, *Lactobacillus* abundance increased substantially over time, whereas *Staphylococcus* abundance peaked at Day 15 before declining. By Day 45, *Lactobacillus* predominated, followed by *Staphylococcus* and *Macrococcus*. The results of a one-way ANOVA showed that group Day 15 had the highest relative abundance of *Staphylococcus* (*P* < 0.05). In parallel, the relative abundance of *Norank_f_norank_o_Chloroplast*, *Norank_f_Mitochondria*, *Pantoea* and *Pseudomonas* at Day 0 was the highest (*P* < 0.05) (Fig. 7(c)).

#### 3.4.3 Differences in bacterial levels during fermentation of yak sour meat

**Fig. 8:**
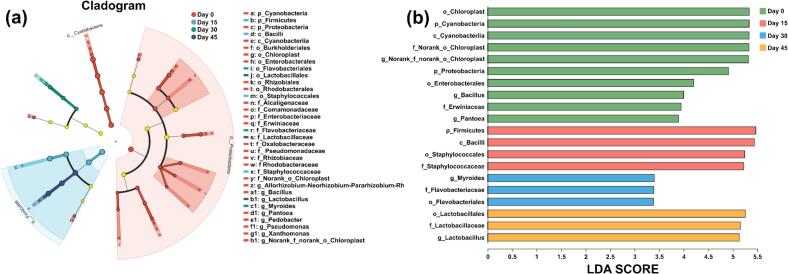
Cladogram plot (a) and bar plot (d) of LEfSe analysis with LDA > 2.

LEfSe analysis was conducted to identify the microbiota features mostly contributing to the differences in bacterial communities during yak sour meat fermentation. (Fig. 8). As shown in Fig. 8 (a-b), there were 20 marker bacteria that gave LDA scores >2 during yak sour meat fermentation. Among them, seven genus-level bacterial taxa were identified as marker bacteria of Day0 group, while *Lactobacillus* was a marker bacterium of Day45 group.

### 3.5 Correlation of bacterial communities with pH and volatile compounds

**Fig. 9:**
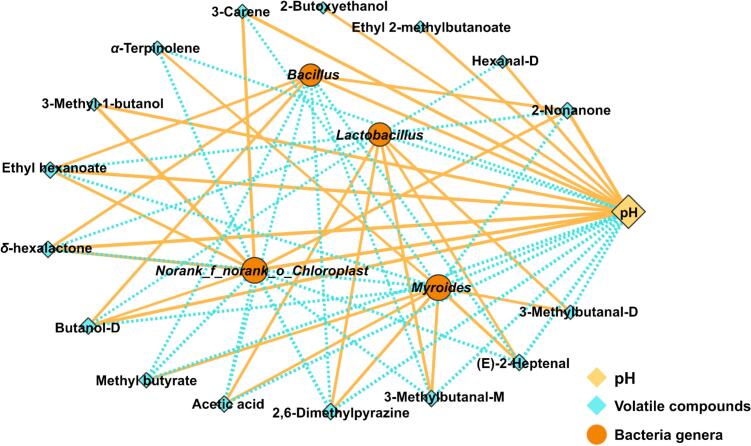
Correlation among volatile compounds, pH and bacterial genera. Yellow and blue lines represent positive and negative correlations. (For interpretation of the references to color in this figure legend, the reader is referred to the web version of this article.)

In this study, we calculated Pearson correlations among bacterial communities, pH and volatile compounds, retaining only strong (|cor| > 0.8) and statistically significant (*P* < 0.05) associations, as shown in Fig. 9. The results showed that the abundance of *Lactobacillus* was correlated positively with acetic acid, (E)-2-heptenal, 3-methylbutanal-D and 3-methylbutanal-M. Moreover, pH was correlated positively with 3-methyl-1-butanol, 2-butoxyethanol, hexanal-D, ethyl 2-methylbutanoate, 2-nonanone, butanol-D, *δ*-hexalactone and ethyl hexanoate.

### 3.6 Analysis of ML

#### 3.6.1 Comparison of model performance based on *E*-nose, E-tongue, and GC-IMS

**Fig. 10:**
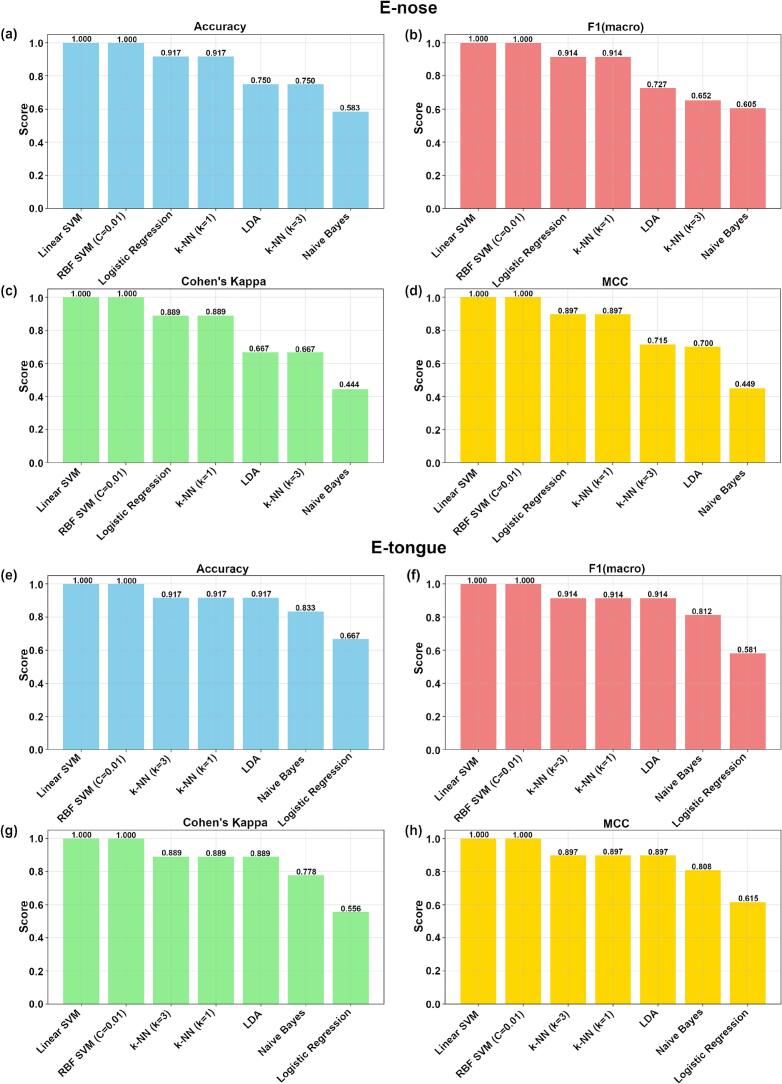
Bar Chart Comparing the Performance of E-nose and E-tongue ML Models.

As shown in Fig. 10 (a-h), various ML models built based on the full features of the E-nose and E-tongue exhibited different classification performance. Overall, SVM models achieved the best results, outperforming logistic regression and k-NN. In contrast, LDA and naive Bayes classifiers consistently demonstrated lower performance across all metrics. Notably, linear SVM and RBF SVM (C = 0.01) both achieved perfect scores (1.000) for accuracy, macro-averaged F1, Cohen's kappa, and the MCC.

**Fig. 11:**
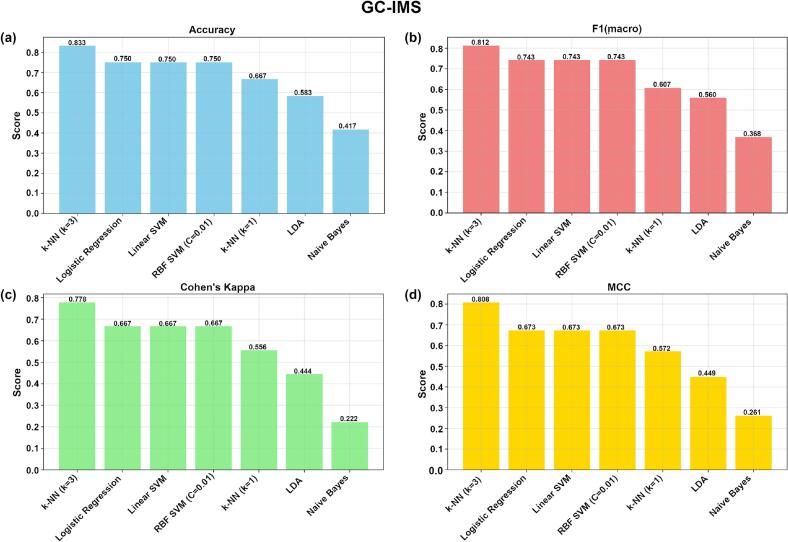
Bar Chart Comparing the Performance of GC-IMS ML Models.

As shown in Figs. 11(a–d), which compare the performance of different ML models on the GC-IMS dataset, model efficacy was evaluated using four metrics: accuracy, macro-averaged F1-score, Cohen's kappa, and the MCC. Among all models, k-NN (k = 3) achieved the highest scores across all metrics, with an accuracy of 0.833, a macro-averaged F1-score of 0.812, Cohen's kappa of 0.778, and an MCC of 0.808—each substantially outperforming the other models compared.

#### 3.6.2 Confusion matrix analysis of optimal models for E-nose, E-tongue, and GC-IMS

**Fig. 12:**
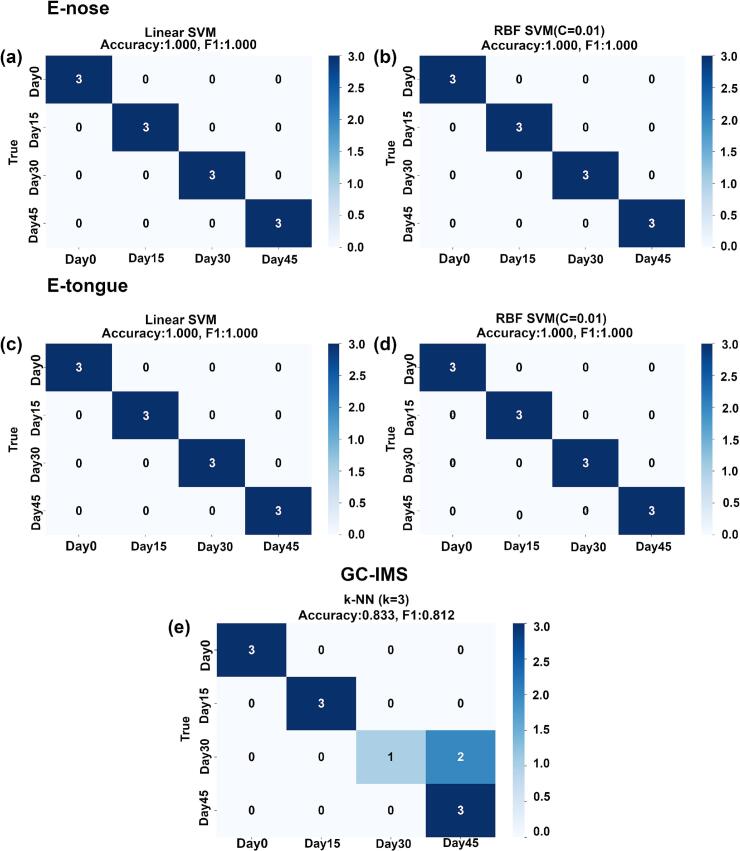
Confusion matrices of discrimination results for yak sour meat fermentation time (Day 0, Day 15, Day 30, and Day 45) using different detection technologies combined with the optimal classification models. (a) Linear SVM model based on e-nose data; (b) RBF SVM model based on E-nose data; (c) Linear SVM model based on E-tongue data; (d) RBF SVM model based on E-tongue data; (e) k-NN (k = 3) model based on GC-IMS data.

There are significant differences in the discrimination results of yak sour meat fermentation times (Day 0, Day 15, Day 30, and Day 45) when combining different detection technologies with optimal classification models. As shown in Figs. 12 (a-d), classification models constructed using electronic nose and electronic tongue based on linear SVM and RBF SVM both achieved accurate identification of all samples. In their confusion matrices, samples of each category were all concentrated along the main diagonal with no misclassification, resulting in overall classification accuracy and F1 values of 1.000. In contrast, as shown in Fig. 12(e), the classification performance of GC-IMS data under the optimal k-NN (k = 3) model was lower compared to the electronic nose and electronic tongue, with an overall accuracy of 0.833 and an F1 value of 0.812. From the confusion matrix, Day 0, Day 15, and Day 45 samples were correctly classified, while 2 Day 30 samples were misclassified as Day 45.

## 4 Discussion

Sour meat is a traditional Chinese fermented product prized for its flavor (Lv et al., 2021; X. Zhao et al., 2023). Yak meat is rich in proteins (20–23%) and unsaturated fatty acids (50–55%) (Z.-X. Fu et al., 2024; Huang et al., 2023). It therefore serves as a nutritious raw material for fermentation. However, research on fermented yak meat products remains limited. Therefore, this study employed an integrated approach combining Intelligent sensory analysis, GC-IMS, and 16S rRNA gene sequencing to investigate bacterial succession during yak meat fermentation and its impact on flavor formation.

The physicochemical characteristics of yak meat appeared to be impacted by fermentation. In detail, the pH significantly decreased from an initial value of 5.59 to 3.98 (*P* < 0.05), mainly driven by fermentative bacterial metabolism of carbohydrates to acids (Flores, 2018). Concurrently, hardness and chewiness increased significantly (*P* < 0.05). This phenomenon likely resulted from pH-dependent protein denaturation, which facilitates structural reorganization through protein aggregation and moisture expulsion (D. Wang et al., 2022).

*E*-tongue and E-nose could successfully discriminate yak sour meat samples collected along fermentation. Half of E-nose sensors exhibited time-dependent responses, while all E-tongue sensors showed statistically significant sensitivity gradients (*P* < 0.05). These results demonstrate the capability of the intelligent sensor system to distinguish aroma and taste characteristics throughout the yak sour meat fermentation process. Unfortunately, while E-tongue and E-nose effectively differentiated fermentation stages based on overall flavor profiles, they lack compound-specific resolution. Therefore, we employed GC-IMS for the untargeted identification of volatile compounds, to precisely characterize the evolution of flavor along fermentation of yak meat.

The 42 volatile compounds identified are predominantly originated from the biochemical pathways involving protein degradation, lipid metabolism, and carbohydrate transformation (Kumar Verma et al., 2022; Cheng et al., 2024). Protein-derived volatiles are generated as endogenous muscle proteases work in tandem with bacterial extracellular proteases to cleave peptide bonds, releasing free amino acids (FAA), then leading to volatile compounds (Grujović et al., 2022). Concurrently, lipid oxidation pathways are initiated by lipolytic enzymes that hydrolyze triglycerides into free fatty acids. These intermediates subsequently undergo *β*-oxidation and Strecker degradation, ultimately producing diverse volatile derivatives (Y. Fu et al., 2022). In addition, carbohydrate metabolism contributes through glycosyl hydrolase activity, where amylases and glycosidases convert polysaccharides into reactive substrates to generate characteristic aldehydes, ketones, and esters.

Esters in fermented yak sour meat form mainly via two pathways. First, carboxylic acids from lipid hydrolysis or proteolysis undergo esterification with alcohols derived from carbohydrates. Second, lipolytic enzymes catalyze the alcoholysis of triglycerides using alcohols (mainly ethanol) from carbohydrates (Huang, Dong, et al., 2022). Esters, like methyl butyrate, ethyl hexanoate, and ethyl 2-methylbutanoate, collectively lead to fruity and sweet aromatic notes, through their characteristic carbonyl‑oxygen bonding structures (He et al., 2022). Notably, cyclic esters such as *δ*-hexalactone exhibit a high flavor persistence, due to their structural stability. *β*-lactones, formed via intramolecular cyclization, play a crucial role in meat flavor development by covalently binding to taste receptors (Dudkiewicz et al., 2022).

Ketones' biosynthesis in meat occurs predominantly through two metabolic pathways: amino acids catabolism and *β*-oxidation of unsaturated fatty acids (Nie et al., 2022). These compounds substantially influence the flavor of the products based on meat, exhibiting higher sensory perception thresholds compared to other lipids oxidation products (Huang, Sun, et al., 2022; Yang, Lan, et al., 2024; B. Zhang et al., 2024). Among them, 2-nonanone is a key volatile that imparts a distinct herbaceous undertone to fermented yak sour meat through controlled off-flavor generation ((Wang et al., 2021, Wu et al., 2023).

As secondary metabolites in lipid oxidation pathways, alcohols are predominantly formed through three biochemical routes: enzymatic conversion of polyunsaturated fatty acids, amino acids deamination, and aldehyde reductase activity (Z. Yang, et al., 2024). These oxygenated compounds enhance flavor complexity by contributing sweet, floral, and fruity notes, as evidenced by their established role in volatile flavor formation (Q. Huang et al., 2022; B. Zhang et al., 2024). 3-methyl-1-butanol generated through *β*-oxidation of fatty acids, exhibiting multimodal aroma characteristics, ranging from caramel sweetness to vegetal earthiness (Kang et al., 2024). Butanol-D, a chiral alcohol, carries distinct stone fruit nuances, suggesting stereochemical specificity in sour meat fermentation. Furthermore, mass spectrometry analysis confirmed 2-butoxyethanol as a beef-specific volatile compound, in agreement with Ozkara's biomarkers identification studies (Ozkara et al., 2019).

Aldehydes in meat products form predominantly through lipid oxidative degradation and amino acid catabolism via Maillard reaction pathways (J. Li et al., 2021). Characterized by exceptionally low sensory thresholds, these compounds critically determine flavor profiles through concentration-dependent effects. Hexanal serves as both a lipid oxidation biomarker and a major contributor to fat-grassy notes at elevated levels (Yin et al., 2021). Concurrently, (E)-2-heptenal is preferentially generated from unsaturated fatty acid oxidation during fermentation processes (Q. Wang et al., 2022), whereas the stereoisomeric 3-methylbutanal (D/M-types) variants exhibit amino acid-dependent biosynthesis patterns (Li, Zhang, et al., 2021). Carbohydrate metabolism by fermentative microbiota serves as the primary route for acetic acid accumulation (Bleicher et al., 2022; Lv, Li, et al., 2019). Pyrazines, heterocyclic nitrogenous compounds, are formed through the Maillard's reaction, involving the interaction of reducing sugars with amino acids (Sun et al., 2022). These substances typically give complex aroma profiles to foods, including nutty, roasted meat and caramel notes (Lee & Kim, 2025). Among pyrazines, 2,6-dimethylpyrazine is particularly significant for the development of distinctive food aromas and flavors. In meat from different sources 2,6-dimethylpyrazine has been identified as a key odor profile component (C. Zhao et al., 2021). While preparing sour meat from yak, the addition of spices represents the main source of terpenes (Ozkara et al., 2019). Among them, *α*-terpinolene gives aromatic pine-like scents complemented by refreshing citrus notes reminiscent of orange and lemon (X. Li et al., 2022; Obiloma et al., 2019). Similarly, 3-carene imparts a complex aroma profile characterized by pine, mint, citrus and camphor, thereby enriching the flavor complexity of yak sour meat (X. Huang et al., 2023).

Fermented meat products derive their distinctive characteristics from dynamic microbial consortia, that determine nutritional profiles, organoleptic properties, and food safety. Bacterial community analysis revealed that *Firmicutes* show a progressive dominance along fermentation, becoming the core bacterial group in yak sour meat. Notably, the samples at Day 0 exhibited a typical predominance of *Cyanobacteria*, then replaced by *Lactobacilli* and *Staphylococci*. On one side, this successional pattern suggests that *Cyanobacteria* function as transient pioneers rather than essential fermenters. On the other side, it suggests that competitive exclusion occurs between oxygen-sensitive fermentative taxa and initial aerobic bacterial group during the maturation of the ecosystem (Wang, Wang, et al., 2021).

At the level of genus, *Norank_f_norank_chloroplast* dominated the community of bacteria during fermentation's initial stages. However, its presence significantly decreased as pH declined (Z. Wang et al., 2020), suggesting that acidification created unfavorable conditions for its survival. Similarly, *Weissella* exhibited a gradual reduction, likely due to competitive exclusion by the rapid proliferation of *Lactobacilli* population (Lin et al., 2022). In contrast, *Staphylococci* initially increased (Day 0–Day 15) but sharply declined thereafter (Day 15–Day 45). This transient growth pattern may result from dual pressures: (1) direct antagonism by *Lactobacillus* through the production of antimicrobial metabolites and (2) progressive pH reduction, which synergistically suppressed *Staphylococcus* proliferation (Xu et al., 2019). Notably, the growth of *Pseudomonas* was strongly inhibited by *Lactobacillus*, particularly under elevated fermentation temperatures. This is particularly interesting, as *Pseudomonas* is a spoilage-associated genus that negatively influences sour meat taste and safety. This suppression is primarily mediated by acidification, as *Lactobacillus*-driven pH reduction creates a hostile environment for spoilage microorganisms. The effective containment of *Pseudomonas* during fermentation therefore enhances both product safety and organoleptic quality (Lv, et al., 2019).

The positive correlation of lactic acid bacteria with acetic acid may be attributed to the association of lactic acid bacteria to the production of organic acids (Dong et al., 2022). Flores et al. have demonstrated that lactic acid bacteria, in meat matrices, are able to synthetize acetic acid through pyruvate and lactate (Flores, 2018). Additionally, *Lactobacillus* levels were positively correlated with aldehyde production during sour meat fermentation, which may result from the bacterium's capacity to release free amino acids and then degrade them to aldehydes (Zang et al., 2022). Focusing on aldehydes, we were able to identify heptanal and butanal, compounds known to enhance the aromatic profile of products created through fermentation (Q. Wang et al., 2022). The pH of the environment during fermentation influences the production of volatile compounds, including ketones, aldehydes, alcohols and esters, likely by modulating bacterial metabolic activity and fatty acid oxidation pathways (Lin et al., 2022). Consequently, pH emerges as a key factor in determining the sensory characteristics of foods based on fermentation.

This study integrates ML with multiple analytical platforms to systematically profile flavor characteristics and achieve precise stage classification in fermented yak sour meat. Classification performance varied significantly across models trained on *E*-nose and E-tongue data. Notably, both linear SVM and RBF SVM (C = 0.01) achieved perfect sample separation, with area under the receiver operating characteristic curve (AUC) values of 1.000—consistent with the established interpretation that AUC closer to 1 indicates higher predictive accuracy (Xiong et al., 2023). These results confirm that SVM-based models can accurately classify flavor profiles from E-nose and E-tongue data. In contrast, when evaluating models on the GC-IMS dataset, k-NN outperformed other classifiers across all metrics, reflecting its stronger nonlinear fitting capacity and greater stability in feature modeling and class discrimination for GC-IMS data, thus delivering balanced and reliable performance in this multiclass task. Interestingly, in the confusion matrix of the optimal GC-IMS model (k-NN, k = 3), two Day-30 samples were misclassified as Day-45. This may be attributed to similar relative concentrations of 3-methylbutanal-M and (E)-2-heptenal in later fermentation stages.

## 5 Conclusion

This study comprehensively elucidated the microbial dynamics and flavor evolution during the fermentation of yak sour meat, using a multi-omics approach and ML framework. The results showed that *Lactobacillus* and *Staphylococcus* dominated the bacterial succession, and that the proliferation of *Lactobacillus* significantly reduced pH and enhanced texture hardness and chewiness, key physicochemical changes determining the structural characteristics of yak sour meat. A total of 42 volatile compounds were identified using GC-IMS, with esters being the main contributors to flavor. Notably, *Lactobacillus* exhibited a strong positive correlation with the synthesis of acetic acid and aldehydes, confirming the central role of microorganisms in flavor formation. E-nose and E-tongue technologies effectively differentiated stage-specific sensory characteristics. The enhanced umami and sweet tastes in the final product directly demonstrate quality improvement during the process. Machine learning models performed well in classifying fermentation stages: the SVM model developed based on electronic nose and electronic tongue data achieved a classification accuracy of 100%, while the k-NN (k = 3) model built on GC-IMS volatile data reached an accuracy of 83.3%. Together, these results validate the reliability of sensor-based classification methods and demonstrate that multi-source data can be effectively used for precise quality evaluation of yak sour meat.

This study integrated metabolomics, metagenomics, intelligent sensing, and machine learning to establish an analytical framework for elucidating the microbe–flavor interaction mechanisms in traditional fermented foods. At the application level, the core microbial strains can be developed into composite starter cultures to standardize the fermentation process, while the sensor- and machine learning-based models can be directly applied for rapid monitoring of fermentation stages. Overall, this study not only deepens the understanding of microbial–flavor interactions in yak sour meat fermentation but also provides a transferable analytical and computational framework for the quality evaluation and industrial optimization of traditional fermented meat products. Future research could be devoted to targeting specific flavor-enhancing strains and to apply them in starter cultures, which potential advancements in industrial production.

## CRediT authorship contribution statement

**Peiting Zhang:** Writing – review & editing, Writing – original draft, Investigation, Formal analysis. **Chenshuo Wang:** Writing – review & editing, Investigation. **Peiyi Wang:** Writing – review & editing. **Zilin Shen:** Writing – review & editing, Investigation. **Jiazhuo Gao:** Writing – review & editing, Investigation. **Shihong Fang:** Writing – review & editing. **Chenglin Zhu:** Writing – review & editing, Writing – original draft, Supervision, Project administration, Funding acquisition, Conceptualization. **Luca Laghi:** Writing – review & editing, Writing – original draft, Methodology.

## Declaration of competing interest

The authors state that they do not have competing financial interests or personal relationships that could have influenced the work presented in this paper.

## Data Availability

Data will be made available on request.

## Appendix A Supplementary data

Supplementary material: E-nose sensors information and molecules characterzied by GC-IMS. Supplementary material
