# Supplementary material for: Unraveling the flavor formation mechanism during yak sour meat fermentation: Integrated multi-omics and machine learning approaches
Source: Food Chem X. 2026 Apr 18;35:103866. doi: 10.1016/j.fochx.2026.103866 (PMC13125153; doi:10.1016/j.fochx.2026.103866)
Supplement: Supplementary file 1 — Supplementary material: E-nose sensors information and molecules characterzied by GC-IMS. [file mmc1.docx]

**Unraveling the flavor formation mechanism during yak sour meat fermentation: Integrated multi-omics and machine learning approaches**

Peiting Zhang^1^, Chenshuo Wang^1^, Peiyi Wang^1^, Zilin Shen^2^, Jiazhuo Gao^2^, Shihong Fang^2^, Chenglin Zhu^[[1]](#footnote-1),3,4 *^, Luca Laghi^5^

Supporting Materials

Table S1. E-nose sensors and its corresponding representative sensitive compounds.

| **Sensors** | **Performance description** |
| --- | --- |
| LY2/LG | Sensitive to oxidizing gas |
| LY2/G | Sensitive to ammonia, carbon monoxide |
| LY2/AA | Sensitive to ethanol |
| LY2/Gh | Sensitive to ammonia/organic amines |
| LY2/gCT1 | Sensitive to hydrogen sulfide |
| LY2/gCT | Sensitive to propane/butane |
| T30/1 | Sensitive to organic solvents |
| P10/1 | Sensitive to hydrocarbons |
| P10/2 | Sensitive to methane |
| P40/1 | Sensitive to fluorine |
| T70/2 | Sensitive to aromatic compounds |
| PA/2 | Sensitive to ethanol, ammonia/organic amines |
| P30/1 | Sensitive to polar compounds (ethanol) |
| P40/2 | Sensitive to heteroatom/chloride/aldehydes |
| P30/2 | Sensitive to alcohol |
| T40/2 | Sensitive to aldehydes |
| T40/1 | Sensitive to chlorinated compounds |
| TA/2 | Sensitive to air quality |

Table S2. Relative content based on GC-IMS characterization (Mean ± SD).

| **Compound Name** | **CAS** | **Molecular Formula** | **RT** | **Day 0** | **Day 15** | **Day 30** | **Day 45** |
| --- | --- | --- | --- | --- | --- | --- | --- |
| Esters |  |  |  |  |  |  |  |
| Ethyl hexanoate-D | 123-66-0 | C_8_H_16_O_2_ | 144.2 | 0.10 ± 1.93×10^-2a^ | 0.13 ± 3.02×10^-2ab^ | 0.11 ± 3.54×10^-2ab^ | 9.84×10^-2^ ± 2.54×10^-2b^ |
| Ethyl hexanoate-M | 123-66-0 | C_8_H_16_O_2_ | 144.2 | 6.44×10^-2^ ± 3.38×10^-3a^ | 6.73×10^-2^ ± 2.68×10^-3a^ | 6.21×10^-2^ ± 3.64×10^-3a^ | 5.58×10^-2^ ± 4.71×10^-3a^ |
| Ethyl 3-methylbutanoate-M | 108-64-5 | C_7_H_14_O_2_ | 130.2 | 7.81×10^-3^ ± 8.26×10^-4a^ | 1.12×10^-2^ ± 7.99×10^-4a^ | 1.08×10^-2^ ± 1.11×10^-3a^ | 1.10×10^-2^ ± 1.93×10^-3a^ |
| Ethyl 3-methylbutanoate-D | 108-64-5 | C_7_H_14_O_2_ | 130.2 | 4.57×10^-3^ ± 8.19×10^-4a^ | 6.36×10^-3^ ± 1.52×10^-3a^ | 4.68×10^-3^ ± 6.71×10^-4a^ | 1.47×10^-2^ ± 1.30×10^-2a^ |
| Ethyl 2-methylbutanoate | 7452-79-1 | C_7_H_14_O_2_ | 130.2 | 3.75×10^-2^ ± 2.10×10^-3a^ | 3.05×10^-2^ ± 1.72×10^-3ab^ | 3.15×10^-2^ ± 4.05×10^-3ab^ | 2.41×10^-2^ ± 1.37×10^-3b^ |
| Methyl butyrate | 623-42-7 | C_5_H_10_O_2_ | 102.1 | 5.85×10^-3^ ± 3.82×10^-4b^ | 4.47×10^-2^ ± 1.61×10^-2a^ | 2.89×10^-2^ ± 2.76×10^-3a^ | 2.65×10^-2^ ± 7.80×10^-3a^ |
| δ-Hexalactone | 823-22-3 | C_6_H_10_O_2_ | 114.1 | 2.12×10^-2^ ± 1.48×10^-3a^ | 1.32×10^-2^ ± 1.86×10^-3ab^ | 1.11×10^-2^ ± 1.97×10^-3b^ | 9.84×10^-3^ ± 3.66×10^-4b^ |
| Pentyl isopentanoate | 25415-62-7 | C_10_H_20_O_2_ | 172.3 | 4.13×10^-3^ ± 2.71×10^-3a^ | 2.19×10^-3^ ± 1.19×10^-3a^ | 3.33×10^-3^ ± 2.10×10^-3a^ | 4.02×10^-3^ ± 1.80×10^-3a^ |
| Ethyl hexanoate | 123-66-0 | C_8_H_16_O_2_ | 144.2 | 5.17×10^-2^ ± 3.07×10^-3a^ | 3.86×10^-2^ ± 2.04×10^-3b^ | 3.79×10^-2^ ± 2.12×10^-3b^ | 3.36×10^-2^ ± 3.23×10^-3b^ |
| Butyl butanoate | 109-21-7 | C_8_H_16_O_2_ | 144.2 | 1.57×10^-2^ ± 1.03×10^-2a^ | 4.82×10^-3^ ± 1.12×10^-3a^ | 4.24×10^-3^ ± 1.25×10^-3a^ | 2.12×10^-3^ ± 5.60×10^-4a^ |
| Methyl heptanoate | 106-73-0 | C_8_H_16_O_2_ | 144.2 | 1.34×10^-2^ ± 8.02×10^-3a^ | 2.02×10^-2^ ± 3.05×10^-3a^ | 1.60×10^-2^ ± 9.61×10^-3a^ | 7.70×10^-3^ ± 5.79×10^-3a^ |
| Ketones |  |  |  |  |  |  |  |
| 3-Hydroxy-2-butanone | 513-86-0 | C_4_H_8_O_2_ | 88.1 | 2.04×10^-3^ ± 1.90×10^-4b^ | 2.67×10^-2^ ± 2.10×10^-2a^ | 1.01×10^-2^ ± 6.41×10^-3ab^ | 2.05×10^-2^ ± 2.08×10^-2a^ |
| 2,3-Pentanedione | 600-14-6 | C_5_H_8_O_2_ | 100.1 | 7.52×10^-3^ ± 3.34×10^-3a^ | 7.25×10^-3^ ± 1.63×10^-3a^ | 9.47×10^-3^ ± 4.20×10^-3a^ | 1.08×10^-2^ ± 1.56×10^-3a^ |
| 2-Nonanone | 821-55-6 | C_9_H_18_O | 142.2 | 3.94×10^-2^ ± 2.81×10^-3a^ | 1.83×10^-2^ ± 2.47×10^-3b^ | 1.50×10^-2^ ± 7.29×10^-4bc^ | 9.96×10^-3^ ± 1.24×10^-3c^ |
| 2-Butanone | 78-93-3 | C_4_H_8_O | 72.1 | 5.18×10^-3^ ± 2.96×10^-3a^ | 2.13×10^-3^ ± 8.01×10^-4a^ | 1.43×10^-3^ ± 2.72×10^-4a^ | 1.61×10^-3^ ± 3.71×10^-4a^ |
| Alcohols |  |  |  |  |  |  |  |
| 3-Methyl-1-butanol | 123-51-3 | C_5_H_12_O | 88.1 | 5.54×10^-2^ ± 2.39×10^-3a^ | 3.64×10^-2^ ± 3.40×10^-3b^ | 3.58×10^-2^ ± 2.39×10^-3b^ | 4.27×10^-2^ ± 2.38×10^-3b^ |
| 3-Methylbutanol-M | 123-51-3 | C_5_H_12_O | 88.1 | 1.52×10^-2^ ± 1.32×10^-3a^ | 1.74×10^-2^ ± 1.73×10^-3a^ | 1.82×10^-2^ ± 2.15×10^-3a^ | 1.86×10^-2^ ± 1.06×10^-3a^ |
| Butanol-M | 71-36-3 | C_4_H_10_O | 74.1 | 4.59×10^-3^ ± 8.52×10^-4a^ | 6.24×10^-3^ ± 1.37×10^-3a^ | 6.13×10^-3^ ± 1.10×10^-3a^ | 6.66×10^-3^ ± 2.93×10^-4a^ |
| Butanol-D | 71-36-3 | C_4_H_10_O | 74.1 | 5.58×10^-2^ ± 2.38×10^-3a^ | 4.16×10^-2^ ± 1.23×10^-3b^ | 4.11×10^-2^ ± 1.15×10^-3b^ | 3.35×10^-2^ ± 4.56×10^-3b^ |
| 2-Methylpropanol-D | 78-83-1 | C_4_H_10_O | 74.1 | 1.97×10^-2^ ± 5.26×10^-3a^ | 6.66×10^-3^ ± 1.48×10^-3b^ | 9.26×10^-3^ ± 4.49×10^-3ab^ | 1.87×10^-2^ ± 3.01×10^-3a^ |
| 2-Methylpropanol-M | 78-83-1 | C_4_H_10_O | 74.1 | 1.31×10^-3^ ± 4.18×10^-4a^ | 1.42×10^-3^ ± 3.75×10^-4a^ | 1.71×10^-3^ ± 5.05×10^-4a^ | 1.91×10^-3^ ± 4.45×10^-4a^ |
| 2-Butoxyethanol | 111-76-2 | C_6_H_14_O_2_ | 118.2 | 1.46×10^-2^ ± 1.14×10^-3a^ | 1.45×10^-2^ ± 2.08×10^-3a^ | 1.09×10^-2^ ± 2.87×10^-3ab^ | 7.63×10^-3^ ± 7.67×10^-4b^ |
| 2-Butanol | 78-92-2 | C_4_H_10_O | 74.1 | 6.63×10^-3^ ± 4.27×10^-3a^ | 4.35×10^-3^ ± 4.47×10^-4a^ | 8.56×10^-3^ ± 6.55×10^-3a^ | 1.27×10^-2^ ± 1.49×10^-3a^ |
| 1-Pentanol | 71-41-0 | C_5_H_12_O | 88.1 | 2.34×10^-3^ ± 1.84×10^-4a^ | 2.67×10^-3^ ± 4.54×10^-4a^ | 2.00×10^-3^ ± 4.36×10^-4a^ | 2.08×10^-3^ ± 1.10×10^-4a^ |
| Aldehydes |  |  |  |  |  |  |  |
| 3-Methylbutanal-M | 590-86-3 | C_5_H_10_O | 86.1 | 2.11×10^-3^ ± 1.12×10^-3b^ | 6.80×10^-3^ ± 1.23×10^-3ab^ | 1.27×10^-2^ ± 7.34×10^-3a^ | 1.74×10^-2^ ± 1.12×10^-3a^ |
| 3-Methylbutanal-D | 590-86-3 | C_5_H_10_O | 86.1 | 1.94×10^-3^ ± 5.50×10^-4b^ | 2.43×10^-3^ ± 1.11×10^-4b^ | 7.77×10^-3^ ± 5.19×10^-3ab^ | 1.20×10^-2^ ± 3.44×10^-3a^ |
| Butyraldehyde | 123-72-8 | C_4_H_8_O | 72.1 | 4.51×10^-2^ ± 5.93×10^-3a^ | 4.09×10^-2^ ± 7.69×10^-3a^ | 4.07×10^-2^ ± 6.35×10^-3a^ | 4.17×10^-2^ ± 4.62×10^-3a^ |
| Hexanal-M | 66-25-1 | C_6_H_12_O | 100.2 | 5.13×10^-3^ ± 1.91×10^-3a^ | 6.04×10^-3^ ± 1.96×10^-3a^ | 6.15×10^-3^ ± 3.22×10^-3a^ | 3.46×10^-3^ ± 2.39×10^-3a^ |
| (E)-2-Heptenal | 18829-55-5 | C_7_H_12_O | 136.2 | 9.64×10^-4^ ± 1.39×10^-4b^ | 2.38×10^-3^ ± 5.51×10^-4ab^ | 3.93×10^-3^ ± 3.71×10^-4a^ | 3.97×10^-3^ ± 7.55×10^-4a^ |
| Hexanal-D | 66-25-1 | C_6_H_12_O | 100.2 | 5.65×10^-2^ ± 3.26×10^-3a^ | 5.65×10^-2^ ± 3.76×10^-3a^ | 3.79×10^-2^ ± 3.99×10^-3b^ | 3.52×10^-2^ ± 6.67×10^-3b^ |
| Others |  |  |  |  |  |  |  |
| β-pinene-M | 127-91-3 | C_10_H_16_ | 136.2 | 4.55×10^-3^ ± 1.66×10^-4a^ | 5.12×10^-3^ ± 4.83×10^-4a^ | 5.55×10^-3^ ± 5.70×10^-4a^ | 4.72×10^-3^ ± 4.32×10^-4a^ |
| β-pinene-D | 127-91-3 | C_10_H_16_ | 136.2 | 2.08×10^-2^ ± 2.92×10^-3a^ | 1.60×10^-2^ ± 1.48×10^-3ab^ | 1.53×10^-2^ ± 3.18×10^-3ab^ | 1.04×10^-2^ ± 2.23×10^-3b^ |
| Camphene | 79-92-5 | C_10_H_16_ | 136.2 | 4.92×10^-3^ ± 1.61×10^-3a^ | 8.08×10^-3^ ± 3.77×10^-3a^ | 7.33×10^-3^ ± 1.47×10^-3a^ | 2.16×10^-2^ ± 1.69×10^-2a^ |
| 2,2,4,6,6-Pentamethylheptane | 13475-82-6 | C_12_H_26_ | 170.3 | 2.26×10^-2^ ± 2.08×10^-2a^ | 1.60×10^-2^ ± 4.82×10^-3a^ | 3.18×10^-2^ ± 2.59×10^-2a^ | 5.48×10^-2^ ± 1.69×10^-2a^ |
| *α*-Terpinolene | 586-62-9 | C_10_H_16_ | 136.2 | 1.32×10^-2^ ± 1.47×10^-3b^ | 3.10×10^-2^ ± 6.33×10^-3a^ | 2.32×10^-2^ ± 6.35×10^-3ab^ | 2.28×10^-2^ ± 4.88×10^-3ab^ |
| Styrene | 100-42-5 | C_8_H_8_ | 104.2 | 3.86×10^-2^ ± 4.78×10^-3a^ | 3.20×10^-2^ ± 2.03×10^-3ab^ | 2.89×10^-2^ ± 3.80×10^-3ab^ | 2.33×10^-2^ ± 2.48×10^-3b^ |
| 3-carene | 13466-78-9 | C_10_H_16_ | 136.2 | 1.68×10^-2^ ± 3.24×10^-3a^ | 9.47×10^-3^ ± 2.38×10^-3ab^ | 6.97×10^-3^ ± 2.79×10^-3b^ | 6.79×10^-3^ ± 1.04×10^-3b^ |
| Acetic acid | 64-19-7 | C_2_H_4_O_2_ | 60.1 | 4.22×10^-2^ ± 2.40×10^-3c^ | 5.70×10^-2^ ± 5.12×10^-3b^ | 9.12×10^-2^ ± 7.84×10^-3ab^ | 0.11 ± 2.87×10^-2a^ |
| 2,6-Dimethylpyrazine | 108-50-9 | C_6_H_8_N_2_ | 108.1 | 1.45×10^-2^ ± 1.83×10^-3b^ | 8.04×10^-2^ ± 1.13×10^-2a^ | 0.12 ± 1.07×10^-2a^ | 9.07×10^-2^ ± 3.19×10^-2a^ |
| 1,3-Diaminopropane | 109-76-2 | C_3_H_10_N_2_ | 74.1 | 2.80×10^-2^ ± 4.80×10^-4b^ | 4.60×10^-2^ ± 4.50×10^-3a^ | 4.68×10^-2^ ± 6.10×10^-3a^ | 3.73×10^-2^ ± 5.67×10^-3a^ |
| Decalin | 91-17-8 | C_10_H_18_ | 138.3 | 2.72×10^-2^ ± 3.28×10^-3a^ | 2.42×10^-2^ ± 1.93×10^-3a^ | 2.52×10^-2^ ± 2.28×10^-3a^ | 2.32×10^-2^ ± 2.61×10^-3a^ |
| Dipropyl disulfide | 629-19-6 | C_6_H_14_S_2_ | 150.3 | 4.07×10^-3^ ± 1.65×10^-3a^ | 1.37×10^-3^ ± 5.56×10^-4a^ | 1.48×10^-3^ ± 4.73×10^-4a^ | 4.20×10^-3^ ± 1.39×10^-3a^ |

The same letter indicating no significant difference.

1. * **Correspondence:** Chenglin Zhu, [chenglin.zhu@swun.edu.cn](mailto:chenglin.zhu@swun.edu.cn), Tel.: +86-028-85928478 [↑](#footnote-ref-1)
